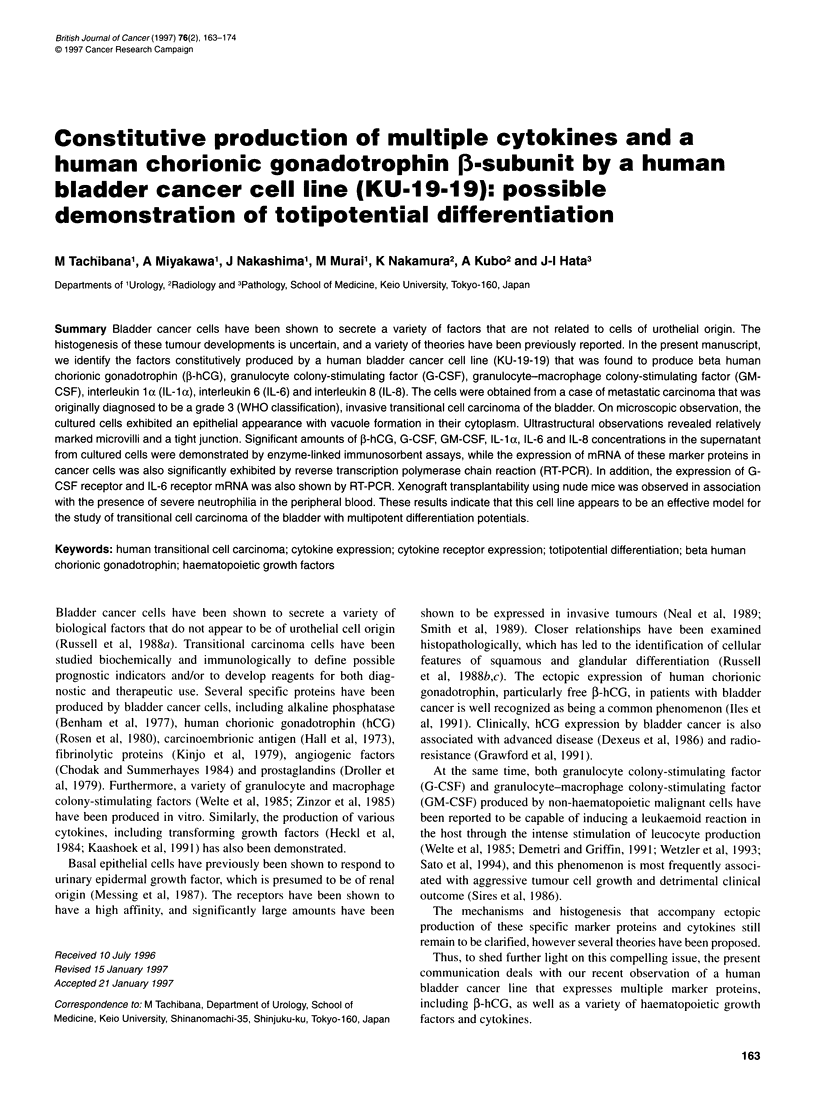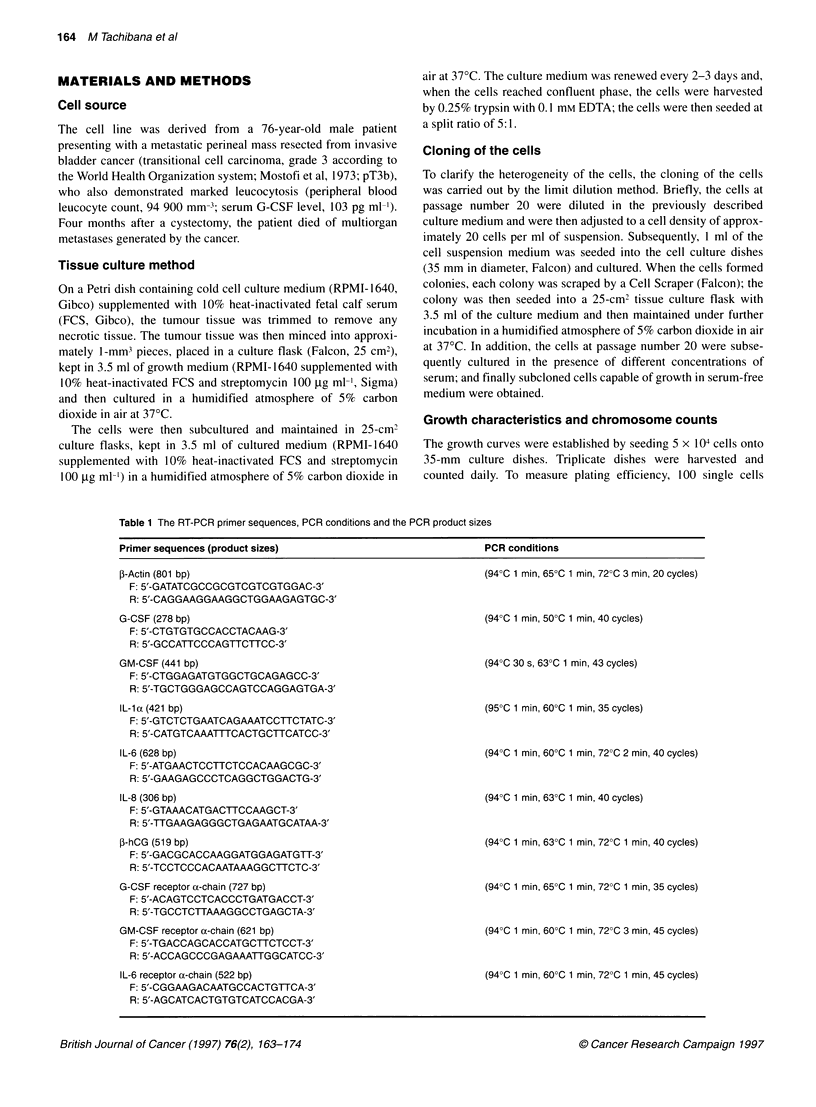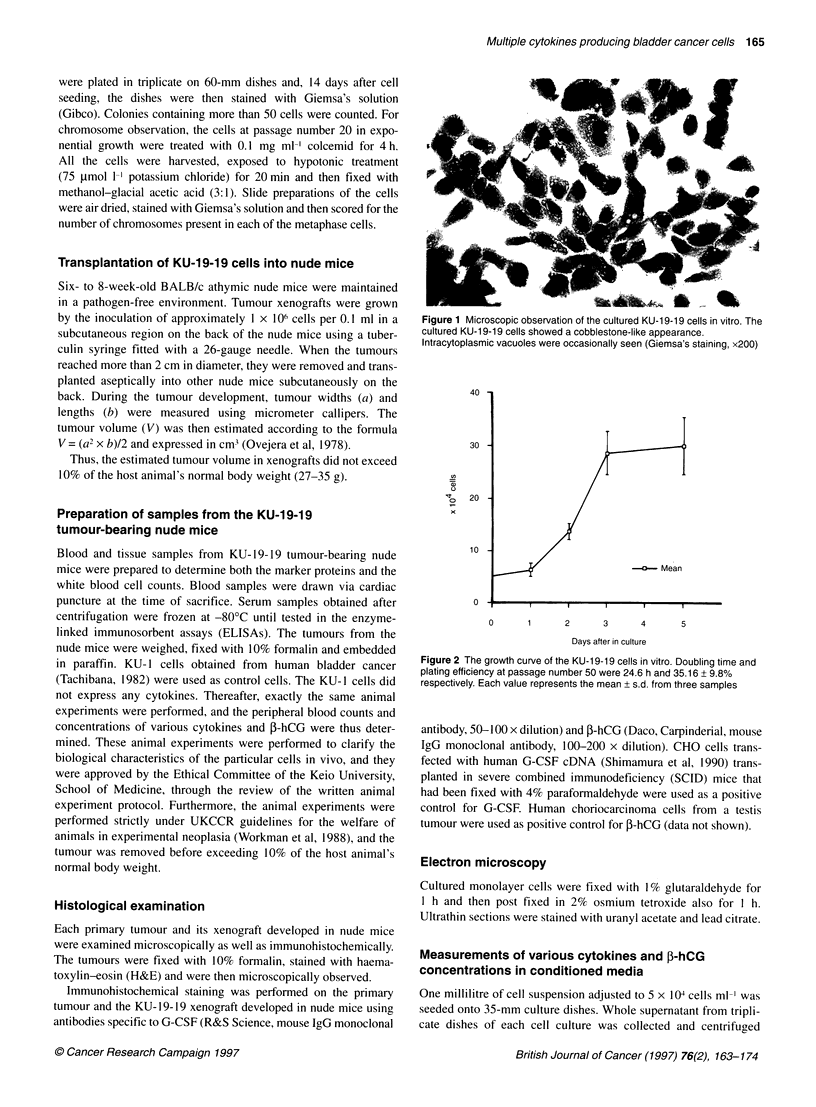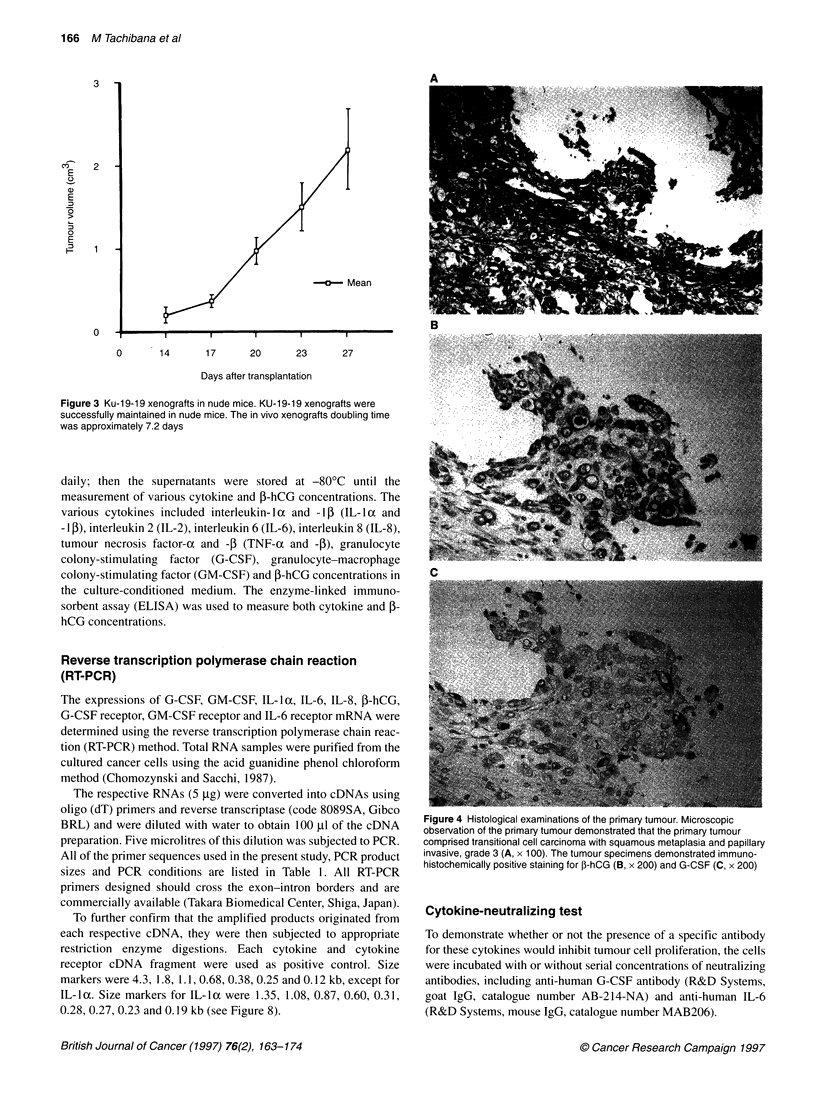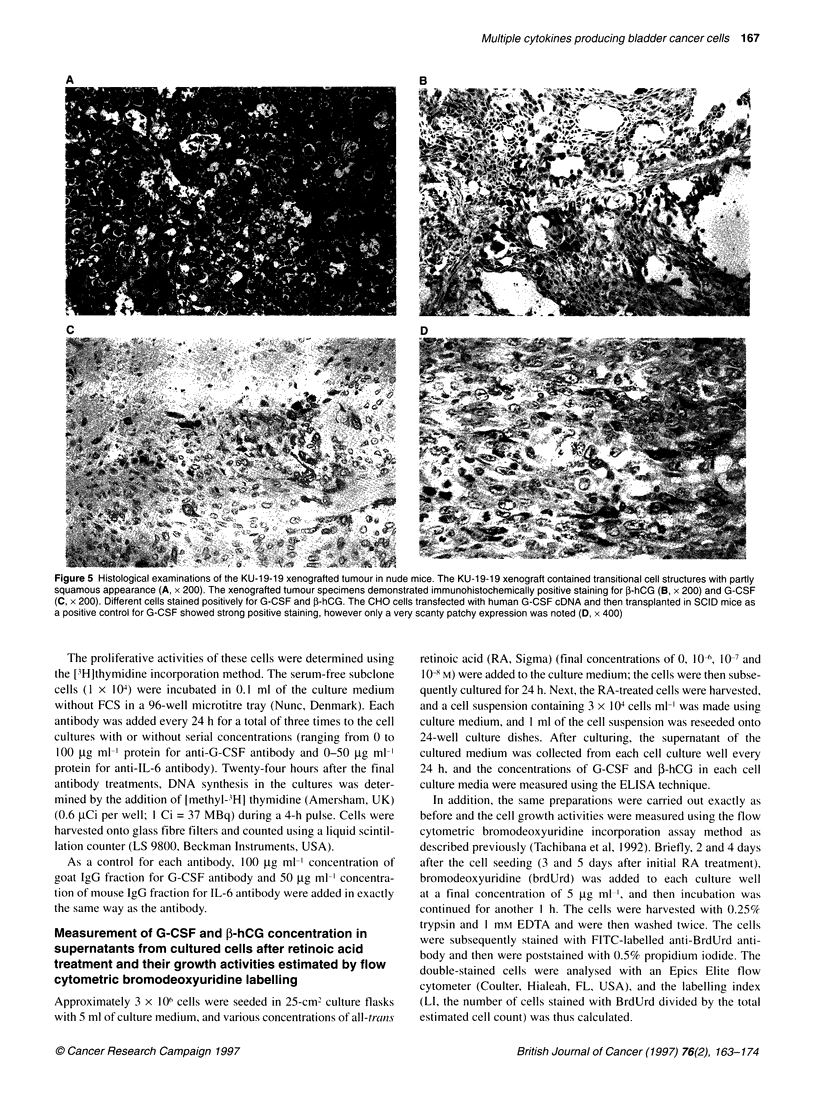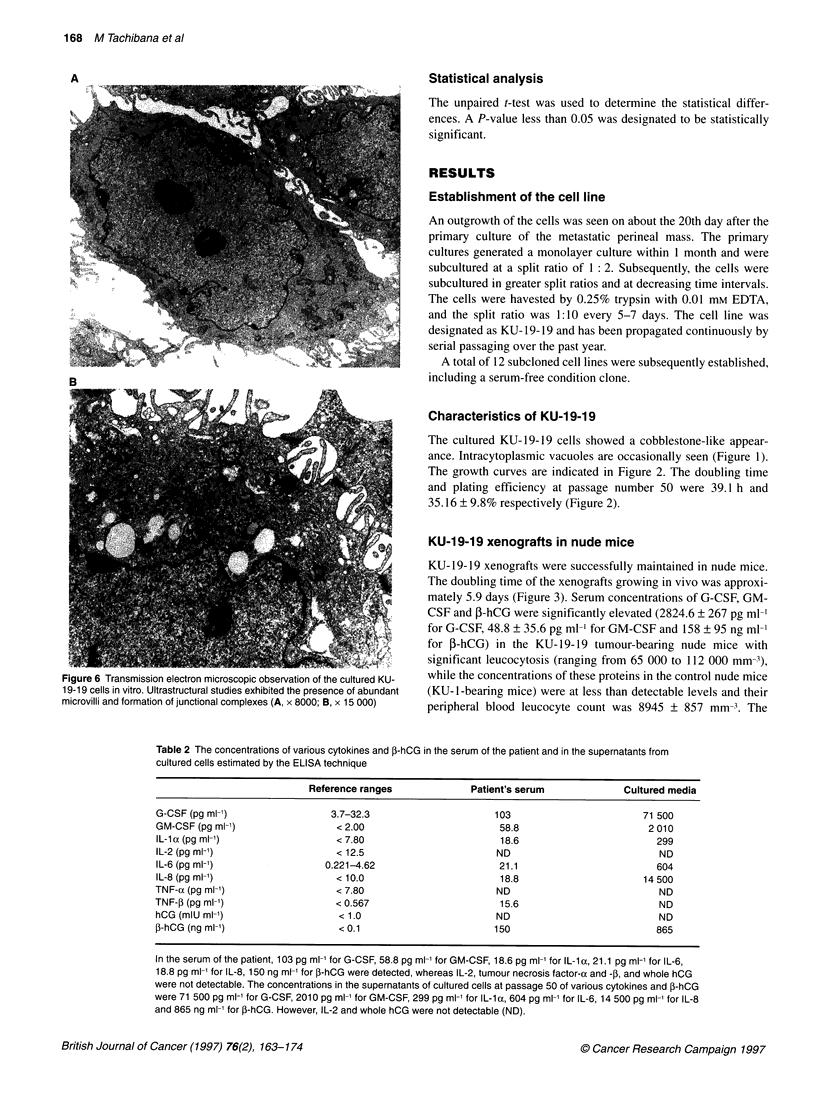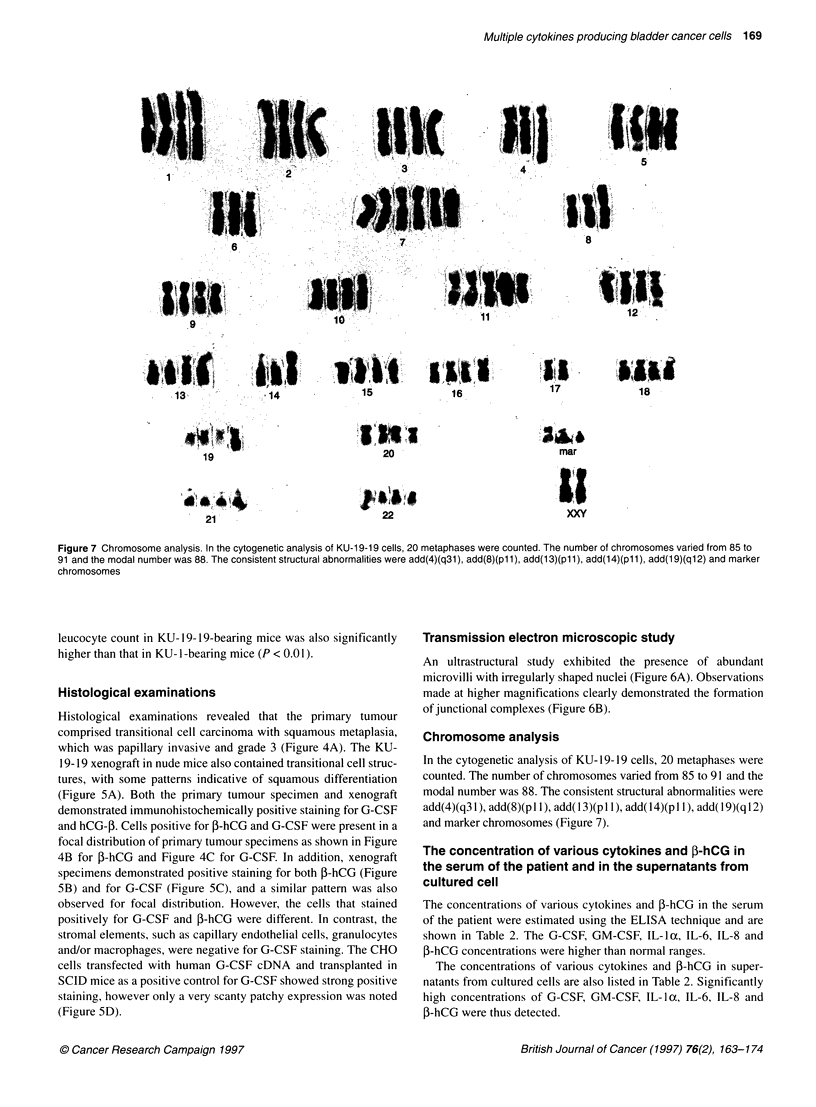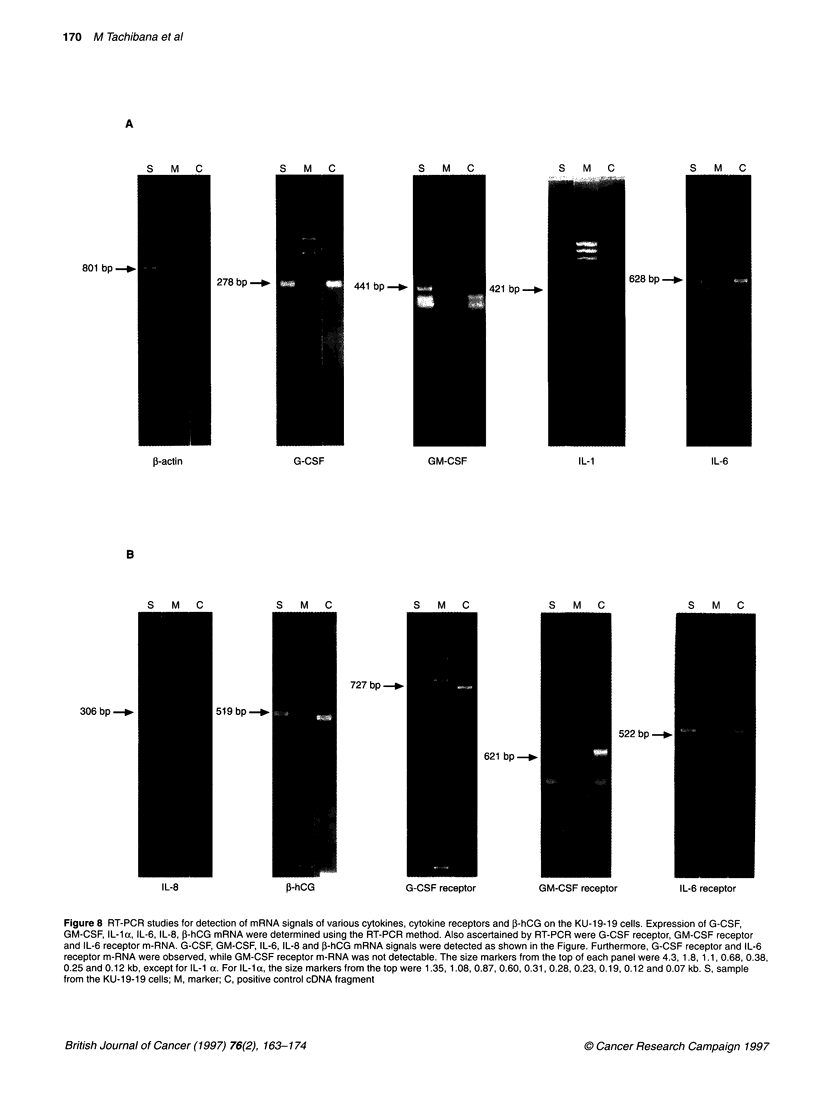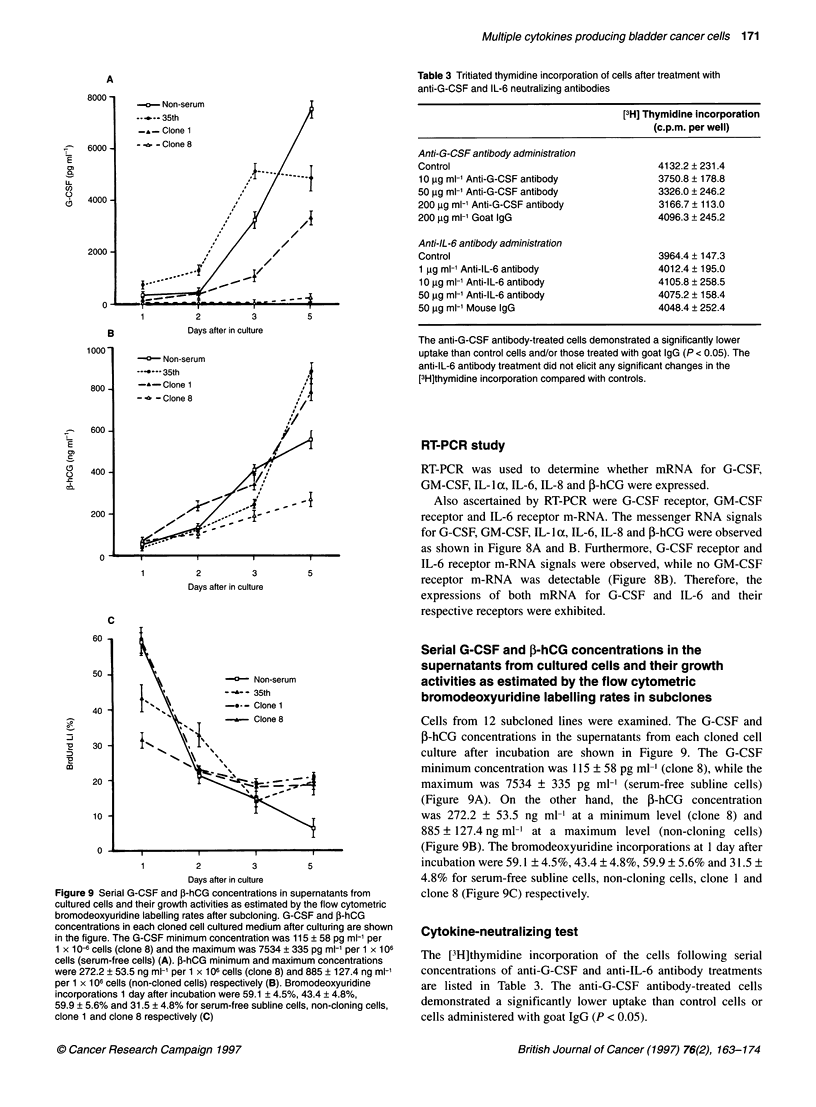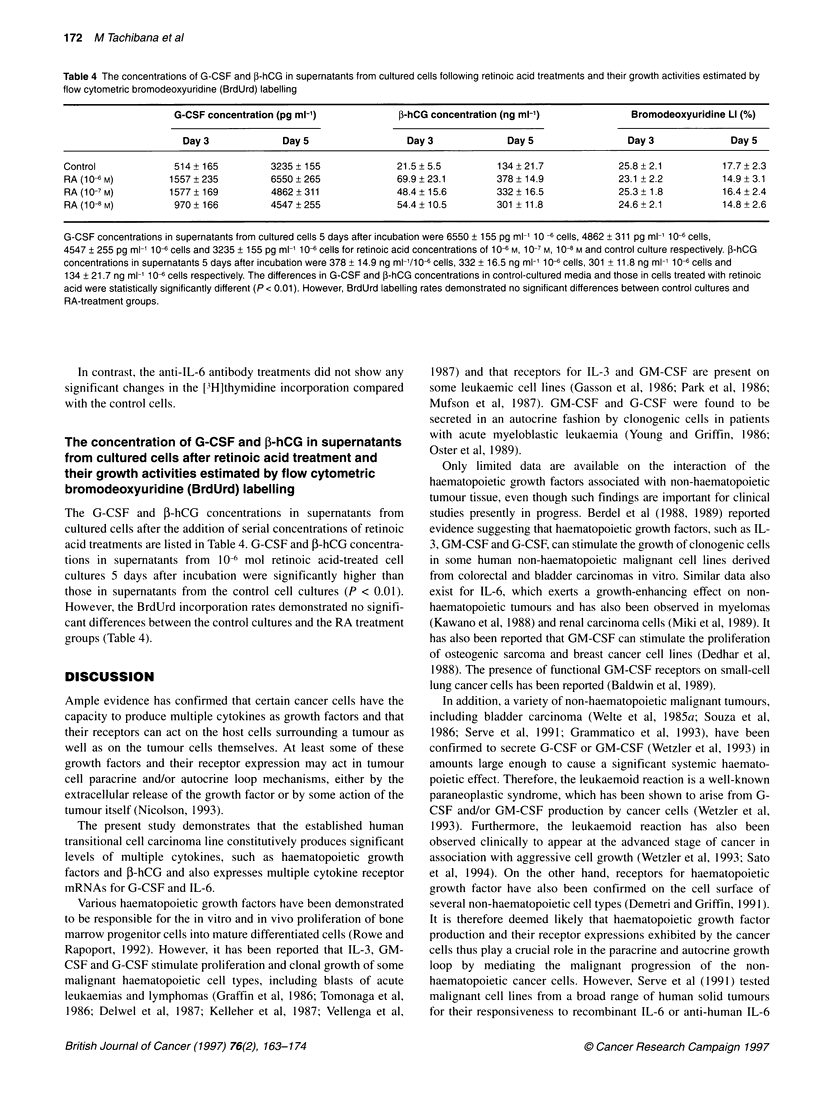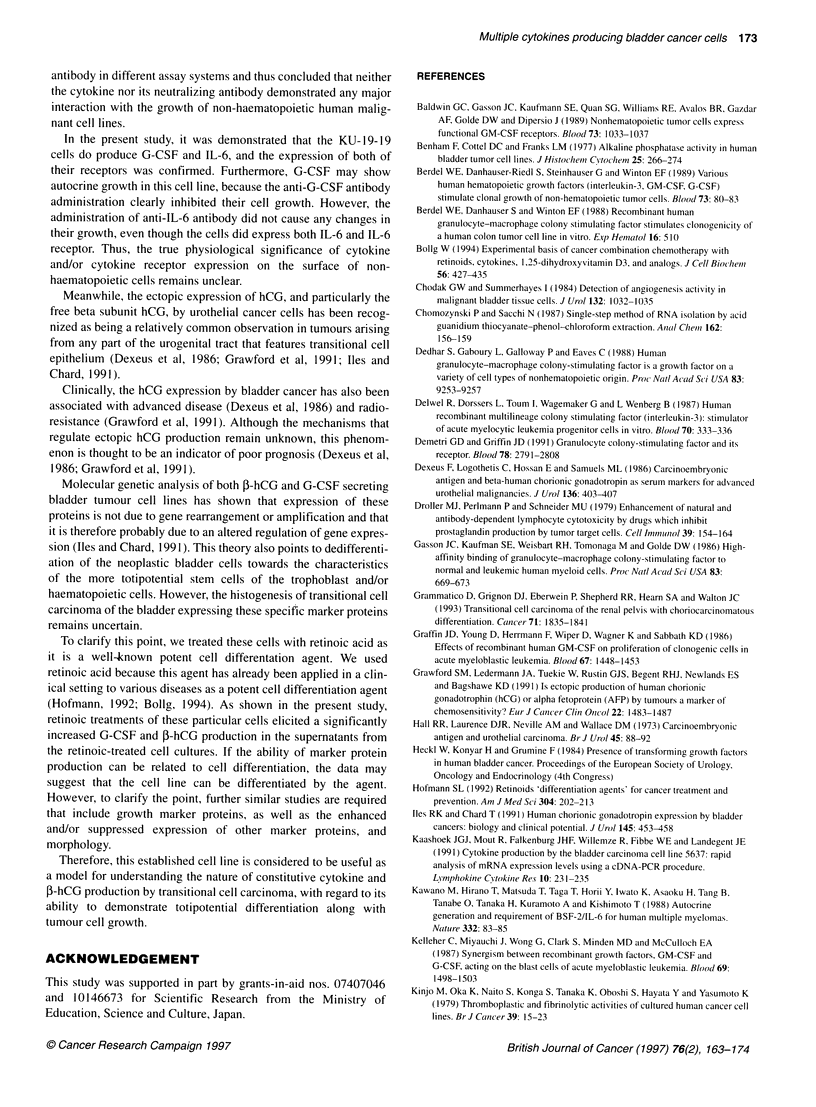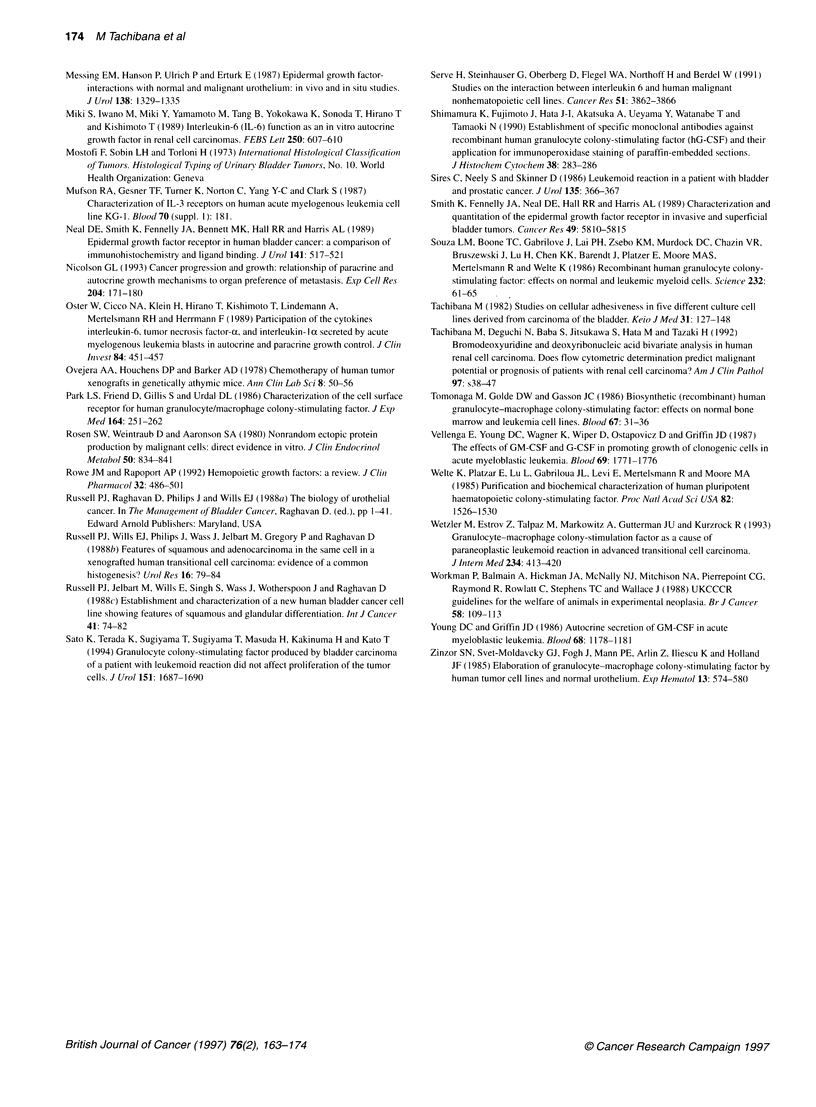# Constitutive production of multiple cytokines and a human chorionic gonadotrophin beta-subunit by a human bladder cancer cell line (KU-19-19): possible demonstration of totipotential differentiation.

**DOI:** 10.1038/bjc.1997.358

**Published:** 1997

**Authors:** M. Tachibana, A. Miyakawa, J. Nakashima, M. Murai, K. Nakamura, A. Kubo, J. I. Hata

**Affiliations:** Department of Urology, School of Medicine, Keio University, Shinjuku-ku, Tokyo, Japan.

## Abstract

**Images:**


British Journal of Cancer (1997) 76(2), 163–174
© 1997 Cancer Research Campaign

### Constitutive production of multiple cytokines and a human chorionic gonadotrophin β-subunit by a human bladder cancer cell line (KU-19-19): possible demonstration of totipotential differentiation

**M Tachibana[1], A Miyakawa[1], J Nakashima[1], M Murai[1], K Nakamura[2], A Kubo[2] and J-I Hata[3]**

Departments of [1]Urology, [2]Radiology and [3]Pathology, School of Medicine, Keio University, Tokyo-160, Japan

**Summary** Bladder cancer cells have been shown to secrete a variety of factors that are not related to cells of urothelial origin. The histogenesis of these tumour developments is uncertain, and a variety of theories have been previously reported. In the present manuscript, we identify the factors constitutively produced by a human bladder cancer cell line (KU-19-19) that was found to produce beta human chorionic gonadotrophin (β-hCG), granulocyte colony-stimulating factor (G-CSF), granulocyte–macrophage colony-stimulating factor (GM-CSF), interleukin 1α (IL-1α), interleukin 6 (IL-6) and interleukin 8 (IL-8). The cells were obtained from a case of metastatic carcinoma that was originally diagnosed to be a grade 3 (WHO classification), invasive transitional cell carcinoma of the bladder. On microscopic observation, the cultured cells exhibited an epithelial appearance with vacuole formation in their cytoplasm. Ultrastructural observations revealed relatively marked microvilli and a tight junction. Significant amounts of β-hCG, G-CSF, GM-CSF, IL-1α, IL-6 and IL-8 concentrations in the supernatant from cultured cells were demonstrated by enzyme-linked immunosorbent assays, while the expression of mRNA of these marker proteins in cancer cells was also significantly exhibited by reverse transcription polymerase chain reaction (RT-PCR). In addition, the expression of G-CSF receptor and IL-6 receptor mRNA was also shown by RT-PCR. Xenograft transplantability using nude mice was observed in association with the presence of severe neutrophilia in the peripheral blood. These results indicate that this cell line appears to be an effective model for the study of transitional cell carcinoma of the bladder with multipotent differentiation potentials.

**Keywords:** human transitional cell carcinoma; cytokine expression; cytokine receptor expression; totipotential differentiation; beta human chorionic gonadotrophin; haematopoietic growth factors

Bladder cancer cells have been shown to secrete a variety of biological factors that do not appear to be of urothelial cell origin (Russell et al, 1988*a*). Transitional carcinoma cells have been studied biochemically and immunologically to define possible prognostic indicators and/or to develop reagents for both diagnostic and therapeutic use. Several specific proteins have been produced by bladder cancer cells, including alkaline phosphatase (Benham et al, 1977), human chorionic gonadotrophin (hCG) (Rosen et al, 1980), carcinoembrionic antigen (Hall et al, 1973), fibrinolytic proteins (Kinjo et al, 1979), angiogenic factors (Chodak and Summerhayes 1984) and prostaglandins (Droller et al, 1979). Furthermore, a variety of granulocyte and macrophage colony-stimulating factors (Welte et al, 1985; Zinzor et al, 1985) have been produced in vitro. Similarly, the production of various cytokines, including transforming growth factors (Heckl et al, 1984; Kaashoek et al, 1991) has also been demonstrated.

Basal epithelial cells have previously been shown to respond to urinary epidermal growth factor, which is presumed to be of renal origin (Messing et al, 1987). The receptors have been shown to have a high affinity, and significantly large amounts have been shown to be expressed in invasive tumours (Neal et al, 1989; Smith et al, 1989). Closer relationships have been examined histopathologically, which has led to the identification of cellular features of squamous and glandular differentiation (Russell et al, 1988*b*,*c*). The ectopic expression of human chorionic gonadotrophin, particularly free β-hCG, in patients with bladder cancer is well recognized as being a common phenomenon (Iles et al, 1991). Clinically, hCG expression by bladder cancer is also associated with advanced disease (Dexeus et al, 1986) and radioresistance (Grawford et al, 1991).

At the same time, both granulocyte colony-stimulating factor (G-CSF) and granulocyte–macrophage colony-stimulating factor (GM-CSF) produced by non-haematopoietic malignant cells have been reported to be capable of inducing a leukaemoid reaction in the host through the intense stimulation of leucocyte production (Welte et al, 1985; Demetri and Griffin, 1991; Wetzler et al, 1993; Sato et al, 1994), and this phenomenon is most frequently associated with aggressive tumour cell growth and detrimental clinical outcome (Sires et al, 1986).

The mechanisms and histogenesis that accompany ectopic production of these specific marker proteins and cytokines still remain to be clarified, however several theories have been proposed.

Thus, to shed further light on this compelling issue, the present communication deals with our recent observation of a human bladder cancer line that expresses multiple marker proteins, including β-hCG, as well as a variety of haematopoietic growth factors and cytokines.

*Received 10 July 1996*
*Revised 15 January 1997*
*Accepted 21 January 1997*

*Correspondence to:* M Tachibana, Department of Urology, School of Medicine, Keio University, Shinanomachi-35, Shinjuku-ku, Tokyo-160, Japan

### MATERIALS AND METHODS

### Cell source

The cell line was derived from a 76-year-old male patient presenting with a metastatic perineal mass resected from invasive bladder cancer (transitional cell carcinoma, grade 3 according to the World Health Organization system; Mostofi et al, 1973; pT3b), who also demonstrated marked leucocytosis (peripheral blood leucocyte count, 94 900 $mm^{-3}$; serum G-CSF level, 103 pg $ml^{-1}$). Four months after a cystectomy, the patient died of multiorgan metastases generated by the cancer.

### Tissue culture method

On a Petri dish containing cold cell culture medium (RPMI-1640, Gibco) supplemented with 10% heat-inactivated fetal calf serum (FCS, Gibco), the tumour tissue was trimmed to remove any necrotic tissue. The tumour tissue was then minced into approximately 1-$mm^3$ pieces, placed in a culture flask (Falcon, 25 $cm^2$), kept in 3.5 ml of growth medium (RPMI-1640 supplemented with 10% heat-inactivated FCS and streptomycin 100 µg $ml^{-1}$, Sigma) and then cultured in a humidified atmosphere of 5% carbon dioxide in air at 37°C.

The cells were then subcultured and maintained in 25-$cm^2$ culture flasks, kept in 3.5 ml of cultured medium (RPMI-1640 supplemented with 10% heat-inactivated FCS and streptomycin 100 µg $ml^{-1}$) in a humidified atmosphere of 5% carbon dioxide in air at 37°C. The culture medium was renewed every 2–3 days and, when the cells reached confluent phase, the cells were harvested by 0.25% trypsin with 0.1 mM EDTA; the cells were then seeded at a split ratio of 5:1.

### Cloning of the cells

To clarify the heterogeneity of the cells, the cloning of the cells was carried out by the limit dilution method. Briefly, the cells at passage number 20 were diluted in the previously described culture medium and were then adjusted to a cell density of approximately 20 cells per ml of suspension. Subsequently, 1 ml of the cell suspension medium was seeded into the cell culture dishes (35 mm in diameter, Falcon) and cultured. When the cells formed colonies, each colony was scraped by a Cell Scraper (Falcon); the colony was then seeded into a 25-$cm^2$ tissue culture flask with 3.5 ml of the culture medium and then maintained under further incubation in a humidified atmosphere of 5% carbon dioxide in air at 37°C. In addition, the cells at passage number 20 were subsequently cultured in the presence of different concentrations of serum; and finally subcloned cells capable of growth in serum-free medium were obtained.

### Growth characteristics and chromosome counts

The growth curves were established by seeding $5 \times 10^4$ cells onto 35-mm culture dishes. Triplicate dishes were harvested and counted daily. To measure plating efficiency, 100 single cells

**Table 1** The RT-PCR primer sequences, PCR conditions and the PCR product sizes

| Primer sequences (product sizes) | PCR conditions |
|---|---|
| β-Actin (801 bp)<br>F: 5′-GATATCGCCGCGTCGTCGTGGAC-3′<br>R: 5′-CAGGAAGGAAGGCTGGAAGAGTGC-3′ | (94°C 1 min, 65°C 1 min, 72°C 3 min, 20 cycles) |
| G-CSF (278 bp)<br>F: 5′-CTGTGTGCCACCTACAAG-3′<br>R: 5′-GCCATTCCCAGTTCTTCC-3′ | (94°C 1 min, 50°C 1 min, 40 cycles) |
| GM-CSF (441 bp)<br>F: 5′-CTGGAGATGTGGCTGCAGAGCC-3′<br>R: 5′-TGCTGGGAGCCAGTCCAGGAGTGA-3′ | (94°C 30 s, 63°C 1 min, 43 cycles) |
| IL-1α (421 bp)<br>F: 5′-GTCTCTGAATCAGAAATCCTTCTATC-3′<br>R: 5′-CATGTCAAATTTCACTGCTTCATCC-3′ | (95°C 1 min, 60°C 1 min, 35 cycles) |
| IL-6 (628 bp)<br>F: 5′-ATGAACTCCTTCTCCACAAGCGC-3′<br>R: 5′-GAAGAGCCCTCAGGCTGGACTG-3′ | (94°C 1 min, 60°C 1 min, 72°C 2 min, 40 cycles) |
| IL-8 (306 bp)<br>F: 5′-GTAAACATGACTTCCAAGCT-3′<br>R: 5′-TTGAAGAGGGCTGAGAATGCATAA-3′ | (94°C 1 min, 63°C 1 min, 40 cycles) |
| β-hCG (519 bp)<br>F: 5′-GACGCACCAAGGATGGAGATGTT-3′<br>R: 5′-TCCTCCCACAATAAAGGCTTCTC-3′ | (94°C 1 min, 63°C 1 min, 72°C 1 min, 40 cycles) |
| G-CSF receptor α-chain (727 bp)<br>F: 5′-ACAGTCCTCACCCTGATGACCT-3′<br>R: 5′-TGCCTCTTAAAGGCCTGAGCTA-3′ | (94°C 1 min, 65°C 1 min, 72°C 1 min, 35 cycles) |
| GM-CSF receptor α-chain (621 bp)<br>F: 5′-TGACCAGCACCATGCTTCTCCT-3′<br>R: 5′-ACCAGCCCGAGAAATTGGCATCC-3′ | (94°C 1 min, 60°C 1 min, 72°C 3 min, 45 cycles) |
| IL-6 receptor α-chain (522 bp)<br>F: 5′-CGGAAGACAATGCCACTGTTCA-3′<br>R: 5′-AGCATCACTGTGTCATCCACGA-3′ | (94°C 1 min, 60°C 1 min, 72°C 1 min, 45 cycles) |

© Cancer Research Campaign 1997

were plated in triplicate on 60-mm dishes and, 14 days after cell seeding, the dishes were then stained with Giemsa's solution (Gibco). Colonies containing more than 50 cells were counted. For chromosome observation, the cells at passage number 20 in exponential growth were treated with 0.1 mg $ml^{-1}$ colcemid for 4 h. All the cells were harvested, exposed to hypotonic treatment (75 µmol $l^{-1}$ potassium chloride) for 20 min and then fixed with methanol–glacial acetic acid (3:1). Slide preparations of the cells were air dried, stained with Giemsa's solution and then scored for the number of chromosomes present in each of the metaphase cells.

### Transplantation of KU-19-19 cells into nude mice

Six- to 8-week-old BALB/c athymic nude mice were maintained in a pathogen-free environment. Tumour xenografts were grown by the inoculation of approximately $1 \times 10^6$ cells per 0.1 ml in a subcutaneous region on the back of the nude mice using a tuberculin syringe fitted with a 26-gauge needle. When the tumours reached more than 2 cm in diameter, they were removed and transplanted aseptically into other nude mice subcutaneously on the back. During the tumour development, tumour widths ($a$) and lengths ($b$) were measured using micrometer callipers. The tumour volume ($V$) was then estimated according to the formula $V = (a^2 \times b)/2$ and expressed in $cm^3$ (Ovejera et al, 1978).

Thus, the estimated tumour volume in xenografts did not exceed 10% of the host animal's normal body weight (27–35 g).

### Preparation of samples from the KU-19-19 tumour-bearing nude mice

Blood and tissue samples from KU-19-19 tumour-bearing nude mice were prepared to determine both the marker proteins and the white blood cell counts. Blood samples were drawn via cardiac puncture at the time of sacrifice. Serum samples obtained after centrifugation were frozen at –80°C until tested in the enzyme-linked immunosorbent assays (ELISAs). The tumours from the nude mice were weighed, fixed with 10% formalin and embedded in paraffin. KU-1 cells obtained from human bladder cancer (Tachibana, 1982) were used as control cells. The KU-1 cells did not express any cytokines. Thereafter, exactly the same animal experiments were performed, and the peripheral blood counts and concentrations of various cytokines and β-hCG were thus determined. These animal experiments were performed to clarify the biological characteristics of the particular cells in vivo, and they were approved by the Ethical Committee of the Keio University, School of Medicine, through the review of the written animal experiment protocol. Furthermore, the animal experiments were performed strictly under UKCCR guidelines for the welfare of animals in experimental neoplasia (Workman et al, 1988), and the tumour was removed before exceeding 10% of the host animal's normal body weight.

### Histological examination

Each primary tumour and its xenograft developed in nude mice were examined microscopically as well as immunohistochemically. The tumours were fixed with 10% formalin, stained with haematoxylin–eosin (H&E) and were then microscopically observed.

Immunohistochemical staining was performed on the primary tumour and the KU-19-19 xenograft developed in nude mice using antibodies specific to G-CSF (R&S Science, mouse IgG monoclonal antibody, 50–100 × dilution) and β-hCG (Daco, Carpinderial, mouse IgG monoclonal antibody, 100–200 × dilution). CHO cells transfected with human G-CSF cDNA (Shimamura et al, 1990) transplanted in severe combined immunodeficiency (SCID) mice that had been fixed with 4% paraformaldehyde were used as a positive control for G-CSF. Human choriocarcinoma cells from a testis tumour were used as positive control for β-hCG (data not shown).

**Figure 1** Microscopic observation of the cultured KU-19-19 cells in vitro. The cultured KU-19-19 cells showed a cobblestone-like appearance. Intracytoplasmic vacuoles were occasionally seen (Giemsa's staining, ×200)

**Figure 2** The growth curve of the KU-19-19 cells in vitro. Doubling time and plating efficiency at passage number 50 were 24.6 h and 35.16 ± 9.8% respectively. Each value represents the mean ± s.d. from three samples

### Electron microscopy

Cultured monolayer cells were fixed with 1% glutaraldehyde for 1 h and then post fixed in 2% osmium tetroxide also for 1 h. Ultrathin sections were stained with uranyl acetate and lead citrate.

### Measurements of various cytokines and β-hCG concentrations in conditioned media

One millilitre of cell suspension adjusted to $5 \times 10^4$ cells $ml^{-1}$ was seeded onto 35-mm culture dishes. Whole supernatant from triplicate dishes of each cell culture was collected and centrifuged

© Cancer Research Campaign 1997

**Figure 3** Ku-19-19 xenografts in nude mice. KU-19-19 xenografts were successfully maintained in nude mice. The in vivo xenografts doubling time was approximately 7.2 days daily; then the supernatants were stored at –80°C until the measurement of various cytokine and β-hCG concentrations. The various cytokines included interleukin-1α and -1β (IL-1α and -1β), interleukin 2 (IL-2), interleukin 6 (IL-6), interleukin 8 (IL-8), tumour necrosis factor-α and -β (TNF-α and -β), granulocyte colony-stimulating factor (G-CSF), granulocyte–macrophage colony-stimulating factor (GM-CSF) and β-hCG concentrations in the culture-conditioned medium. The enzyme-linked immunosorbent assay (ELISA) was used to measure both cytokine and β-hCG concentrations.

### Reverse transcription polymerase chain reaction (RT-PCR)

The expressions of G-CSF, GM-CSF, IL-1α, IL-6, IL-8, β-hCG, G-CSF receptor, GM-CSF receptor and IL-6 receptor mRNA were determined using the reverse transcription polymerase chain reaction (RT-PCR) method. Total RNA samples were purified from the cultured cancer cells using the acid guanidine phenol chloroform method (Chomozynski and Sacchi, 1987).

The respective RNAs (5 μg) were converted into cDNAs using oligo (dT) primers and reverse transcriptase (code 8089SA, Gibco BRL) and were diluted with water to obtain 100 μl of the cDNA preparation. Five microlitres of this dilution was subjected to PCR. All of the primer sequences used in the present study, PCR product sizes and PCR conditions are listed in Table 1. All RT-PCR primers designed should cross the exon–intron borders and are commercially available (Takara Biomedical Center, Shiga, Japan).

To further confirm that the amplified products originated from each respective cDNA, they were then subjected to appropriate restriction enzyme digestions. Each cytokine and cytokine receptor cDNA fragment were used as positive control. Size markers were 4.3, 1.8, 1.1, 0.68, 0.38, 0.25 and 0.12 kb, except for IL-1α. Size markers for IL-1α were 1.35, 1.08, 0.87, 0.60, 0.31, 0.28, 0.27, 0.23 and 0.19 kb (see Figure 8).

**Figure 4** Histological examinations of the primary tumour. Microscopic observation of the primary tumour demonstrated that the primary tumour comprised transitional cell carcinoma with squamous metaplasia and papillary invasive, grade 3 (**A**, × 100). The tumour specimens demonstrated immunohistochemically positive staining for β-hCG (**B**, × 200) and G-CSF (**C**, × 200)

### Cytokine-neutralizing test

To demonstrate whether or not the presence of a specific antibody for these cytokines would inhibit tumour cell proliferation, the cells were incubated with or without serial concentrations of neutralizing antibodies, including anti-human G-CSF antibody (R&D Systems, goat IgG, catalogue number AB-214-NA) and anti-human IL-6 (R&D Systems, mouse IgG, catalogue number MAB206).

© Cancer Research Campaign 1997

**Figure 5** Histological examinations of the KU-19-19 xenografted tumour in nude mice. The KU-19-19 xenograft contained transitional cell structures with partly squamous appearance (**A**, × 200). The xenografted tumour specimens demonstrated immunohistochemically positive staining for β-hCG (**B**, × 200) and G-CSF (**C**, × 200). Different cells stained positively for G-CSF and β-hCG. The CHO cells transfected with human G-CSF cDNA and then transplanted in SCID mice as a positive control for G-CSF showed strong positive staining, however only a very scanty patchy expression was noted (**D**, × 400)

The proliferative activities of these cells were determined using the [$^3$H]thymidine incorporation method. The serum-free subclone cells ($1 \times 10^4$) were incubated in 0.1 ml of the culture medium without FCS in a 96-well microtitre tray (Nunc, Denmark). Each antibody was added every 24 h for a total of three times to the cell cultures with or without serial concentrations (ranging from 0 to 100 μg ml$^{-1}$ protein for anti-G-CSF antibody and 0–50 μg ml$^{-1}$ protein for anti-IL-6 antibody). Twenty-four hours after the final antibody treatments, DNA synthesis in the cultures was determined by the addition of [methyl-$^3$H] thymidine (Amersham, UK) (0.6 μCi per well; 1 Ci = 37 MBq) during a 4-h pulse. Cells were harvested onto glass fibre filters and counted using a liquid scintillation counter (LS 9800, Beckman Instruments, USA).

As a control for each antibody, 100 μg ml$^{-1}$ concentration of goat IgG fraction for G-CSF antibody and 50 μg ml$^{-1}$ concentration of mouse IgG fraction for IL-6 antibody were added in exactly the same way as the antibody.

### Measurement of G-CSF and β-hCG concentration in supernatants from cultured cells after retinoic acid treatment and their growth activities estimated by flow cytometric bromodeoxyuridine labelling

Approximately $3 \times 10^6$ cells were seeded in 25-cm$^2$ culture flasks with 5 ml of culture medium, and various concentrations of all-*trans* retinoic acid (RA, Sigma) (final concentrations of 0, $10^{-6}$, $10^{-7}$ and $10^{-8}$ M) were added to the culture medium; the cells were then subsequently cultured for 24 h. Next, the RA-treated cells were harvested, and a cell suspension containing $3 \times 10^4$ cells ml$^{-1}$ was made using culture medium, and 1 ml of the cell suspension was reseeded onto 24-well culture dishes. After culturing, the supernatant of the cultured medium was collected from each cell culture well every 24 h, and the concentrations of G-CSF and β-hCG in each cell culture media were measured using the ELISA technique.

In addition, the same preparations were carried out exactly as before and the cell growth activities were measured using the flow cytometric bromodeoxyuridine incorporation assay method as described previously (Tachibana et al, 1992). Briefly, 2 and 4 days after the cell seeding (3 and 5 days after initial RA treatment), bromodeoxyuridine (brdUrd) was added to each culture well at a final concentration of 5 μg ml$^{-1}$, and then incubation was continued for another 1 h. The cells were harvested with 0.25% trypsin and 1 mM EDTA and were then washed twice. The cells were subsequently stained with FITC-labelled anti-BrdUrd antibody and then were poststained with 0.5% propidium iodide. The double-stained cells were analysed with an Epics Elite flow cytometer (Coulter, Hialeah, FL, USA), and the labelling index (LI, the number of cells stained with BrdUrd divided by the total estimated cell count) was thus calculated.

© Cancer Research Campaign 1997

**Figure 6** Transmission electron microscopic observation of the cultured KU-19-19 cells in vitro. Ultrastructural studies exhibited the presence of abundant microvilli and formation of junctional complexes (**A**, × 8000; **B**, × 15 000)

### Statistical analysis

The unpaired *t*-test was used to determine the statistical differences. A *P*-value less than 0.05 was designated to be statistically significant.

### RESULTS

### Establishment of the cell line

An outgrowth of the cells was seen on about the 20th day after the primary culture of the metastatic perineal mass. The primary cultures generated a monolayer culture within 1 month and were subcultured at a split ratio of 1 : 2. Subsequently, the cells were subcultured in greater split ratios and at decreasing time intervals. The cells were havested by 0.25% trypsin with 0.01 mM EDTA, and the split ratio was 1:10 every 5–7 days. The cell line was designated as KU-19-19 and has been propagated continuously by serial passaging over the past year.

A total of 12 subcloned cell lines were subsequently established, including a serum-free condition clone.

### Characteristics of KU-19-19

The cultured KU-19-19 cells showed a cobblestone-like appearance. Intracytoplasmic vacuoles are occasionally seen (Figure 1). The growth curves are indicated in Figure 2. The doubling time and plating efficiency at passage number 50 were 39.1 h and 35.16 ± 9.8% respectively (Figure 2).

### KU-19-19 xenografts in nude mice

KU-19-19 xenografts were successfully maintained in nude mice. The doubling time of the xenografts growing in vivo was approximately 5.9 days (Figure 3). Serum concentrations of G-CSF, GM-CSF and β-hCG were significantly elevated (2824.6 ± 267 pg $ml^{-1}$ for G-CSF, 48.8 ± 35.6 pg $ml^{-1}$ for GM-CSF and 158 ± 95 ng $ml^{-1}$ for β-hCG) in the KU-19-19 tumour-bearing nude mice with significant leucocytosis (ranging from 65 000 to 112 000 $mm^{-3}$), while the concentrations of these proteins in the control nude mice (KU-1-bearing mice) were at less than detectable levels and their peripheral blood leucocyte count was 8945 ± 857 $mm^{-3}$. The

**Table 2** The concentrations of various cytokines and β-hCG in the serum of the patient and in the supernatants from cultured cells estimated by the ELISA technique

| | Reference ranges | Patient's serum | Cultured media |
|---|---|---|---|
| G-CSF (pg $ml^{-1}$) | 3.7–32.3 | 103 | 71 500 |
| GM-CSF (pg $ml^{-1}$) | < 2.00 | 58.8 | 2 010 |
| IL-1α (pg $ml^{-1}$) | < 7.80 | 18.6 | 299 |
| IL-2 (pg $ml^{-1}$) | < 12.5 | ND | ND |
| IL-6 (pg $ml^{-1}$) | 0.221–4.62 | 21.1 | 604 |
| IL-8 (pg $ml^{-1}$) | < 10.0 | 18.8 | 14 500 |
| TNF-α (pg $ml^{-1}$) | < 7.80 | ND | ND |
| TNF-β (pg $ml^{-1}$) | < 0.567 | 15.6 | ND |
| hCG (mIU $ml^{-1}$) | < 1.0 | ND | ND |
| β-hCG (ng $ml^{-1}$) | < 0.1 | 150 | 865 |

In the serum of the patient, 103 pg $ml^{-1}$ for G-CSF, 58.8 pg $ml^{-1}$ for GM-CSF, 18.6 pg $ml^{-1}$ for IL-1α, 21.1 pg $ml^{-1}$ for IL-6, 18.8 pg $ml^{-1}$ for IL-8, 150 ng $ml^{-1}$ for β-hCG were detected, whereas IL-2, tumour necrosis factor-α and -β, and whole hCG were not detectable. The concentrations in the supernatants of cultured cells at passage 50 of various cytokines and β-hCG were 71 500 pg $ml^{-1}$ for G-CSF, 2010 pg $ml^{-1}$ for GM-CSF, 299 pg $ml^{-1}$ for IL-1α, 604 pg $ml^{-1}$ for IL-6, 14 500 pg $ml^{-1}$ for IL-8 and 865 ng $ml^{-1}$ for β-hCG. However, IL-2 and whole hCG were not detectable (ND).

© Cancer Research Campaign 1997

**Figure 7** Chromosome analysis. In the cytogenetic analysis of KU-19-19 cells, 20 metaphases were counted. The number of chromosomes varied from 85 to 91 and the modal number was 88. The consistent structural abnormalities were add(4)(q31), add(8)(p11), add(13)(p11), add(14)(p11), add(19)(q12) and marker chromosomes leucocyte count in KU-19-19-bearing mice was also significantly higher than that in KU-1-bearing mice ($P < 0.01$).

### Histological examinations

Histological examinations revealed that the primary tumour comprised transitional cell carcinoma with squamous metaplasia, which was papillary invasive and grade 3 (Figure 4A). The KU-19-19 xenograft in nude mice also contained transitional cell structures, with some patterns indicative of squamous differentiation (Figure 5A). Both the primary tumour specimen and xenograft demonstrated immunohistochemically positive staining for G-CSF and hCG-β. Cells positive for β-hCG and G-CSF were present in a focal distribution of primary tumour specimens as shown in Figure 4B for β-hCG and Figure 4C for G-CSF. In addition, xenograft specimens demonstrated positive staining for both β-hCG (Figure 5B) and for G-CSF (Figure 5C), and a similar pattern was also observed for focal distribution. However, the cells that stained positively for G-CSF and β-hCG were different. In contrast, the stromal elements, such as capillary endothelial cells, granulocytes and/or macrophages, were negative for G-CSF staining. The CHO cells transfected with human G-CSF cDNA and transplanted in SCID mice as a positive control for G-CSF showed strong positive staining, however only a very scanty patchy expression was noted (Figure 5D).

### Transmission electron microscopic study

An ultrastructural study exhibited the presence of abundant microvilli with irregularly shaped nuclei (Figure 6A). Observations made at higher magnifications clearly demonstrated the formation of junctional complexes (Figure 6B).

### Chromosome analysis

In the cytogenetic analysis of KU-19-19 cells, 20 metaphases were counted. The number of chromosomes varied from 85 to 91 and the modal number was 88. The consistent structural abnormalities were add(4)(q31), add(8)(p11), add(13)(p11), add(14)(p11), add(19)(q12) and marker chromosomes (Figure 7).

### The concentration of various cytokines and β-hCG in the serum of the patient and in the supernatants from cultured cell

The concentrations of various cytokines and β-hCG in the serum of the patient were estimated using the ELISA technique and are shown in Table 2. The G-CSF, GM-CSF, IL-1α, IL-6, IL-8 and β-hCG concentrations were higher than normal ranges.

The concentrations of various cytokines and β-hCG in supernatants from cultured cells are also listed in Table 2. Significantly high concentrations of G-CSF, GM-CSF, IL-1α, IL-6, IL-8 and β-hCG were thus detected.

© Cancer Research Campaign 1997

**Figure 8** RT-PCR studies for detection of mRNA signals of various cytokines, cytokine receptors and β-hCG on the KU-19-19 cells. Expression of G-CSF, GM-CSF, IL-1α, IL-6, IL-8, β-hCG mRNA were determined using the RT-PCR method. Also ascertained by RT-PCR were G-CSF receptor, GM-CSF receptor and IL-6 receptor m-RNA. G-CSF, GM-CSF, IL-6, IL-8 and β-hCG mRNA signals were detected as shown in the Figure. Furthermore, G-CSF receptor and IL-6 receptor m-RNA were observed, while GM-CSF receptor m-RNA was not detectable. The size markers from the top of each panel were 4.3, 1.8, 1.1, 0.68, 0.38, 0.25 and 0.12 kb, except for IL-1 α. For IL-1α, the size markers from the top were 1.35, 1.08, 0.87, 0.60, 0.31, 0.28, 0.23, 0.19, 0.12 and 0.07 kb. S, sample from the KU-19-19 cells; M, marker; C, positive control cDNA fragment

 © Cancer Research Campaign 1997

**Figure 9** Serial G-CSF and β-hCG concentrations in supernatants from cultured cells and their growth activities as estimated by the flow cytometric bromodeoxyuridine labelling rates after subcloning. G-CSF and β-hCG concentrations in each cloned cell cultured medium after culturing are shown in the figure. The G-CSF minimum concentration was 115 ± 58 pg $ml^{-1}$ per $1 \times 10^{-6}$ cells (clone 8) and the maximum was 7534 ± 335 pg $ml^{-1}$ per $1 \times 10^{6}$ cells (serum-free cells) (**A**). β-hCG minimum and maximum concentrations were 272.2 ± 53.5 ng $ml^{-1}$ per $1 \times 10^{6}$ cells (clone 8) and 885 ± 127.4 ng $ml^{-1}$ per $1 \times 10^{6}$ cells (non-cloned cells) respectively (**B**). Bromodeoxyuridine incorporations 1 day after incubation were 59.1 ± 4.5%, 43.4 ± 4.8%, 59.9 ± 5.6% and 31.5 ± 4.8% for serum-free subline cells, non-cloning cells, clone 1 and clone 8 respectively (**C**)

**Table 3** Tritiated thymidine incorporation of cells after treatment with anti-G-CSF and IL-6 neutralizing antibodies

| | [$^3$H] Thymidine incorporation (c.p.m. per well) |
|---|---|
| *Anti-G-CSF antibody administration* | |
| Control | 4132.2 ± 231.4 |
| 10 μg $ml^{-1}$ Anti-G-CSF antibody | 3750.8 ± 178.8 |
| 50 μg $ml^{-1}$ Anti-G-CSF antibody | 3326.0 ± 246.2 |
| 200 μg $ml^{-1}$ Anti-G-CSF antibody | 3166.7 ± 113.0 |
| 200 μg $ml^{-1}$ Goat IgG | 4096.3 ± 245.2 |
| *Anti-IL-6 antibody administration* | |
| Control | 3964.4 ± 147.3 |
| 1 μg $ml^{-1}$ Anti-IL-6 antibody | 4012.4 ± 195.0 |
| 10 μg $ml^{-1}$ Anti-IL-6 antibody | 4105.8 ± 258.5 |
| 50 μg $ml^{-1}$ Anti-IL-6 antibody | 4075.2 ± 158.4 |
| 50 μg $ml^{-1}$ Mouse IgG | 4048.4 ± 252.4 |

The anti-G-CSF antibody-treated cells demonstrated a significantly lower uptake than control cells and/or those treated with goat IgG ($P < 0.05$). The anti-IL-6 antibody treatment did not elicit any significant changes in the [$^3$H]thymidine incorporation compared with controls.

### RT-PCR study

RT-PCR was used to determine whether mRNA for G-CSF, GM-CSF, IL-1α, IL-6, IL-8 and β-hCG were expressed.

Also ascertained by RT-PCR were G-CSF receptor, GM-CSF receptor and IL-6 receptor m-RNA. The messenger RNA signals for G-CSF, GM-CSF, IL-1α, IL-6, IL-8 and β-hCG were observed as shown in Figure 8A and B. Furthermore, G-CSF receptor and IL-6 receptor m-RNA signals were observed, while no GM-CSF receptor m-RNA was detectable (Figure 8B). Therefore, the expressions of both mRNA for G-CSF and IL-6 and their respective receptors were exhibited.

### Serial G-CSF and β-hCG concentrations in the supernatants from cultured cells and their growth activities as estimated by the flow cytometric bromodeoxyuridine labelling rates in subclones

Cells from 12 subcloned lines were examined. The G-CSF and β-hCG concentrations in the supernatants from each cloned cell culture after incubation are shown in Figure 9. The G-CSF minimum concentration was 115 ± 58 pg $ml^{-1}$ (clone 8), while the maximum was 7534 ± 335 pg $ml^{-1}$ (serum-free subline cells) (Figure 9A). On the other hand, the β-hCG concentration was 272.2 ± 53.5 ng $ml^{-1}$ at a minimum level (clone 8) and 885 ± 127.4 ng $ml^{-1}$ at a maximum level (non-cloning cells) (Figure 9B). The bromodeoxyuridine incorporations at 1 day after incubation were 59.1 ± 4.5%, 43.4 ± 4.8%, 59.9 ± 5.6% and 31.5 ± 4.8% for serum-free subline cells, non-cloning cells, clone 1 and clone 8 (Figure 9C) respectively.

### Cytokine-neutralizing test

The [$^3$H]thymidine incorporation of the cells following serial concentrations of anti-G-CSF and anti-IL-6 antibody treatments are listed in Table 3. The anti-G-CSF antibody-treated cells demonstrated a significantly lower uptake than control cells or cells administered with goat IgG ($P < 0.05$).

© Cancer Research Campaign 1997

**Table 4** The concentrations of G-CSF and β-hCG in supernatants from cultured cells following retinoic acid treatments and their growth activities estimated by flow cytometric bromodeoxyuridine (BrdUrd) labelling

| | G-CSF concentration (pg ml$^{-1}$) | | β-hCG concentration (ng ml$^{-1}$) | | Bromodeoxyuridine LI (%) | |
|---|---|---|---|---|---|---|
| | Day 3 | Day 5 | Day 3 | Day 5 | Day 3 | Day 5 |
| Control | 514 ± 165 | 3235 ± 155 | 21.5 ± 5.5 | 134 ± 21.7 | 25.8 ± 2.1 | 17.7 ± 2.3 |
| RA ($10^{-6}$ M) | 1557 ± 235 | 6550 ± 265 | 69.9 ± 23.1 | 378 ± 14.9 | 23.1 ± 2.2 | 14.9 ± 3.1 |
| RA ($10^{-7}$ M) | 1577 ± 169 | 4862 ± 311 | 48.4 ± 15.6 | 332 ± 16.5 | 25.3 ± 1.8 | 16.4 ± 2.4 |
| RA ($10^{-8}$ M) | 970 ± 166 | 4547 ± 255 | 54.4 ± 10.5 | 301 ± 11.8 | 24.6 ± 2.1 | 14.8 ± 2.6 |

G-CSF concentrations in supernatants from cultured cells 5 days after incubation were 6550 ± 155 pg ml$^{-1}$ $10^{-6}$ cells, 4862 ± 311 pg ml$^{-1}$ $10^{-6}$ cells, 4547 ± 255 pg ml$^{-1}$ $10^{-6}$ cells and 3235 ± 155 pg ml$^{-1}$ $10^{-6}$ cells for retinoic acid concentrations of $10^{-6}$ M, $10^{-7}$ M, $10^{-8}$ M and control culture respectively. β-hCG concentrations in supernatants 5 days after incubation were 378 ± 14.9 ng ml$^{-1}$/$10^{-6}$ cells, 332 ± 16.5 ng ml$^{-1}$ $10^{-6}$ cells, 301 ± 11.8 ng ml$^{-1}$ $10^{-6}$ cells and 134 ± 21.7 ng ml$^{-1}$ $10^{-6}$ cells respectively. The differences in G-CSF and β-hCG concentrations in control-cultured media and those in cells treated with retinoic acid were statistically significantly different ($P < 0.01$). However, BrdUrd labelling rates demonstrated no significant differences between control cultures and RA-treatment groups.

In contrast, the anti-IL-6 antibody treatments did not show any significant changes in the [$^3$H]thymidine incorporation compared with the control cells.

### The concentration of G-CSF and β-hCG in supernatants from cultured cells after retinoic acid treatment and their growth activities estimated by flow cytometric bromodeoxyuridine (BrdUrd) labelling

The G-CSF and β-hCG concentrations in supernatants from cultured cells after the addition of serial concentrations of retinoic acid treatments are listed in Table 4. G-CSF and β-hCG concentrations in supernatants from $10^{-6}$ mol retinoic acid-treated cell cultures 5 days after incubation were significantly higher than those in supernatants from the control cell cultures ($P < 0.01$). However, the BrdUrd incorporation rates demonstrated no significant differences between the control cultures and the RA treatment groups (Table 4).

### DISCUSSION

Ample evidence has confirmed that certain cancer cells have the capacity to produce multiple cytokines as growth factors and that their receptors can act on the host cells surrounding a tumour as well as on the tumour cells themselves. At least some of these growth factors and their receptor expression may act in tumour cell paracrine and/or autocrine loop mechanisms, either by the extracellular release of the growth factor or by some action of the tumour itself (Nicolson, 1993).

The present study demonstrates that the established human transitional cell carcinoma line constitutively produces significant levels of multiple cytokines, such as haematopoietic growth factors and β-hCG and also expresses multiple cytokine receptor mRNAs for G-CSF and IL-6.

Various haematopoietic growth factors have been demonstrated to be responsible for the in vitro and in vivo proliferation of bone marrow progenitor cells into mature differentiated cells (Rowe and Rapoport, 1992). However, it has been reported that IL-3, GM-CSF and G-CSF stimulate proliferation and clonal growth of some malignant haematopoietic cell types, including blasts of acute leukaemias and lymphomas (Graffin et al, 1986; Tomonaga et al, 1986; Delwel et al, 1987; Kelleher et al, 1987; Vellenga et al, 1987) and that receptors for IL-3 and GM-CSF are present on some leukaemic cell lines (Gasson et al, 1986; Park et al, 1986; Mufson et al, 1987). GM-CSF and G-CSF were found to be secreted in an autocrine fashion by clonogenic cells in patients with acute myeloblastic leukaemia (Young and Griffin, 1986; Oster et al, 1989).

Only limited data are available on the interaction of the haematopoietic growth factors associated with non-haematopoietic tumour tissue, even though such findings are important for clinical studies presently in progress. Berdel et al (1988, 1989) reported evidence suggesting that haematopoietic growth factors, such as IL-3, GM-CSF and G-CSF, can stimulate the growth of clonogenic cells in some human non-haematopoietic malignant cell lines derived from colorectal and bladder carcinomas in vitro. Similar data also exist for IL-6, which exerts a growth-enhancing effect on non-haematopoietic tumours and has also been observed in myelomas (Kawano et al, 1988) and renal carcinoma cells (Miki et al, 1989). It has also been reported that GM-CSF can stimulate the proliferation of osteogenic sarcoma and breast cancer cell lines (Dedhar et al, 1988). The presence of functional GM-CSF receptors on small-cell lung cancer cells has been reported (Baldwin et al, 1989).

In addition, a variety of non-haematopoietic malignant tumours, including bladder carcinoma (Welte et al, 1985*a*; Souza et al, 1986; Serve et al, 1991; Grammatico et al, 1993), have been confirmed to secrete G-CSF or GM-CSF (Wetzler et al, 1993) in amounts large enough to cause a significant systemic haematopoietic effect. Therefore, the leukaemoid reaction is a well-known paraneoplastic syndrome, which has been shown to arise from G-CSF and/or GM-CSF production by cancer cells (Wetzler et al, 1993). Furthermore, the leukaemoid reaction has also been observed clinically to appear at the advanced stage of cancer in association with aggressive cell growth (Wetzler et al, 1993; Sato et al, 1994). On the other hand, receptors for haematopoietic growth factor have also been confirmed on the cell surface of several non-haematopoietic cell types (Demetri and Griffin, 1991). It is therefore deemed likely that haematopoietic growth factor production and their receptor expressions exhibited by the cancer cells thus play a crucial role in the paracrine and autocrine growth loop by mediating the malignant progression of the non-haematopoietic cancer cells. However, Serve et al (1991) tested malignant cell lines from a broad range of human solid tumours for their responsiveness to recombinant IL-6 or anti-human IL-6

 © Cancer Research Campaign 1997

antibody in different assay systems and thus concluded that neither the cytokine nor its neutralizing antibody demonstrated any major interaction with the growth of non-haematopoietic human malignant cell lines.

In the present study, it was demonstrated that the KU-19-19 cells do produce G-CSF and IL-6, and the expression of both of their receptors was confirmed. Furthermore, G-CSF may show autocrine growth in this cell line, because the anti-G-CSF antibody administration clearly inhibited their cell growth. However, the administration of anti-IL-6 antibody did not cause any changes in their growth, even though the cells did express both IL-6 and IL-6 receptor. Thus, the true physiological significance of cytokine and/or cytokine receptor expression on the surface of non-haematopoietic cells remains unclear.

Meanwhile, the ectopic expression of hCG, and particularly the free beta subunit hCG, by urothelial cancer cells has been recognized as being a relatively common observation in tumours arising from any part of the urogenital tract that features transitional cell epithelium (Dexeus et al, 1986; Grawford et al, 1991; Iles and Chard, 1991).

Clinically, the hCG expression by bladder cancer has also been associated with advanced disease (Dexeus et al, 1986) and radio-resistance (Grawford et al, 1991). Although the mechanisms that regulate ectopic hCG production remain unknown, this phenomenon is thought to be an indicator of poor prognosis (Dexeus et al, 1986; Grawford et al, 1991).

Molecular genetic analysis of both β-hCG and G-CSF secreting bladder tumour cell lines has shown that expression of these proteins is not due to gene rearrangement or amplification and that it is therefore probably due to an altered regulation of gene expression (Iles and Chard, 1991). This theory also points to dedifferentiation of the neoplastic bladder cells towards the characteristics of the more totipotential stem cells of the trophoblast and/or haematopoietic cells. However, the histogenesis of transitional cell carcinoma of the bladder expressing these specific marker proteins remains uncertain.

To clarify this point, we treated these cells with retinoic acid as it is a well-known potent cell differentation agent. We used retinoic acid because this agent has already been applied in a clinical setting to various diseases as a potent cell differentiation agent (Hofmann, 1992; Bollg, 1994). As shown in the present study, retinoic treatments of these particular cells elicited a significantly increased G-CSF and β-hCG production in the supernatants from the retinoic-treated cell cultures. If the ability of marker protein production can be related to cell differentiation, the data may suggest that the cell line can be differentiated by the agent. However, to clarify the point, further similar studies are required that include growth marker proteins, as well as the enhanced and/or suppressed expression of other marker proteins, and morphology.

Therefore, this established cell line is considered to be useful as a model for understanding the nature of constitutive cytokine and β-hCG production by transitional cell carcinoma, with regard to its ability to demonstrate totipotential differentiation along with tumour cell growth.

### ACKNOWLEDGEMENT

This study was supported in part by grants-in-aid nos. 07407046 and 10146673 for Scientific Research from the Ministry of Education, Science and Culture, Japan.

### REFERENCES

Baldwin GC, Gasson JC, Kaufmann SE, Quan SG, Williams RE, Avalos BR, Gazdar AF, Golde DW and Dipersio J (1989) Nonhematopoietic tumor cells express functional GM-CSF receptors. *Blood* **73**: 1033–1037

Benham F, Cottel DC and Franks LM (1977) Alkaline phosphatase activity in human bladder tumor cell lines. *J Histochem Cytochem* **25**: 266–274

Berdel WE, Danhauser-Riedl S, Steinhauser G and Winton EF (1989) Various human hematopoietic growth factors (interleukin-3, GM-CSF, G-CSF) stimulate clonal growth of non-hematopoietic tumor cells. *Blood* **73**: 80–83

Berdel WE, Danhauser S and Winton EF (1988) Recombinant human granulocyte–macrophage colony stimulating factor stimulates clonogenicity of a human colon tumor cell line in vitro. *Exp Hematol* **16**: 510

Bollg W (1994) Experimental basis of cancer combination chemotherapy with retinoids, cytokines, 1,25-dihydroxyvitamin D3, and analogs. *J Cell Biochem* **56**: 427–435

Chodak GW and Summerhayes I (1984) Detection of angiogenesis activity in malignant bladder tissue cells. *J Urol* **132**: 1032–1035

Chomozynski P and Sacchi N (1987) Single-step method of RNA isolation by acid guanidium thiocyanate–phenol–chloroform extraction. *Anal Chem* **162**: 156–159

Dedhar S, Gaboury L, Galloway P and Eaves C (1988) Human granulocyte–macrophage colony-stimulating factor is a growth factor on a variety of cell types of nonhematopoietic origin. *Proc Natl Acad Sci USA* **83**: 9253–9257

Delwel R, Dorssers L, Toum I, Wagemaker G and L Wenberg B (1987) Human recombinant multilineage colony stimulating factor (interleukin-3): stimulator of acute myelocytic leukemia progenitor cells in vitro. *Blood* **70**: 333–336

Demetri GD and Griffin JD (1991) Granulocyte colony-stimulating factor and its receptor. *Blood* **78**: 2791–2808

Dexeus F, Logothetis C, Hossan E and Samuels ML (1986) Carcinoembryonic antigen and beta-human chorionic gonadotropin as serum markers for advanced urothelial malignancies. *J Urol* **136**: 403–407

Droller MJ, Perlmann P and Schneider MU (1979) Enhancement of natural and antibody-dependent lymphocyte cytotoxicity by drugs which inhibit prostaglandin production by tumor target cells. *Cell Immunol* **39**: 154–164

Gasson JC, Kaufman SE, Weisbart RH, Tomonaga M and Golde DW (1986) High-affinity binding of granulocyte–macrophage colony-stimulating factor to normal and leukemic human myeloid cells. *Proc Natl Acad Sci USA* **83**: 669–673

Grammatico D, Grignon DJ, Eberwein P, Shepherd RR, Hearn SA and Walton JC (1993) Transitional cell carcinoma of the renal pelvis with choriocarcinomatous differentiation. *Cancer* **71**: 1835–1841

Graffin JD, Young D, Herrmann F, Wiper D, Wagner K and Sabbath KD (1986) Effects of recombinant human GM-CSF on proliferation of clonogenic cells in acute myeloblastic leukemia. *Blood* **67**: 1448–1453

Grawford SM, Ledermann JA, Tuekie W, Rustin GJS, Begent RHJ, Newlands ES and Bagshawe KD (1991) Is ectopic production of human chorionic gonadotrophin (hCG) or alpha fetoprotein (AFP) by tumours a marker of chemosensitivity? *Eur J Cancer Clin Oncol* **22**: 1483–1487

Hall RR, Laurence DJR, Neville AM and Wallace DM (1973) Carcinoembryonic antigen and urothelial carcinoma. *Br J Urol* **45**: 88–92

Heckl W, Konyar H and Grumine F (1984) Presence of transforming growth factors in human bladder cancer. Proceedings of the European Society of Urology, Oncology and Endocrinology (4th Congress)

Hofmann SL (1992) Retinoids 'differentiation agents' for cancer treatment and prevention. *Am J Med Sci* **304**: 202–213

Iles RK and Chard T (1991) Human chorionic gonadotropin expression by bladder cancers: biology and clinical potential. *J Urol* **145**: 453–458

Kaashoek JGJ, Mout R, Falkenburg JHF, Willemze R, Fibbe WE and Landegent JE (1991) Cytokine production by the bladder carcinoma cell line 5637: rapid analysis of mRNA expression levels using a cDNA-PCR procedure. *Lymphokine Cytokine Res* **10**: 231–235

Kawano M, Hirano T, Matsuda T, Taga T, Horii Y, Iwato K, Asaoku H, Tang B, Tanabe O, Tanaka H, Kuramoto A and Kishimoto T (1988) Autocrine generation and requirement of BSF-2/IL-6 for human multiple myelomas. *Nature* **332**: 83–85

Kelleher C, Miyauchi J, Wong G, Clark S, Minden MD and McCulloch EA (1987) Synergism between recombinant growth factors, GM-CSF and G-CSF, acting on the blast cells of acute myeloblastic leukemia. *Blood* **69**: 1498–1503

Kinjo M, Oka K, Naito S, Konga S, Tanaka K, Oboshi S, Hayata Y and Yasumoto K (1979) Thromboplastic and fibrinolytic activities of cultured human cancer cell lines. *Br J Cancer* **39**: 15–23

© Cancer Research Campaign 1997 

Messing EM, Hanson P, Ulrich P and Erturk E (1987) Epidermal growth factor-interactions with normal and malignant urothelium: in vivo and in situ studies. *J Urol* **138**: 1329–1335

Miki S, Iwano M, Miki Y, Yamamoto M, Tang B, Yokokawa K, Sonoda T, Hirano T and Kishimoto T (1989) Interleukin-6 (IL-6) function as an in vitro autocrine growth factor in renal cell carcinomas. *FEBS Lett* **250**: 607–610

Mostofi F, Sobin LH and Torloni H (1973) *International Histological Classification of Tumors. Histological Typing of Urinary Bladder Tumors*, No. 10. World Health Organization: Geneva

Mufson RA, Gesner TF, Turner K, Norton C, Yang Y-C and Clark S (1987) Characterization of IL-3 receptors on human acute myelogenous leukemia cell line KG-1. *Blood* **70** (suppl. 1): 181.

Neal DE, Smith K, Fennelly JA, Bennett MK, Hall RR and Harris AL (1989) Epidermal growth factor receptor in human bladder cancer: a comparison of immunohistochemistry and ligand binding. *J Urol* **141**: 517–521

Nicolson GL (1993) Cancer progression and growth: relationship of paracrine and autocrine growth mechanisms to organ preference of metastasis. *Exp Cell Res* **204**: 171–180

Oster W, Cicco NA, Klein H, Hirano T, Kishimoto T, Lindemann A, Mertelsmann RH and Herrmann F (1989) Participation of the cytokines interleukin-6, tumor necrosis factor-α, and interleukin-1α secreted by acute myelogenous leukemia blasts in autocrine and paracrine growth control. *J Clin Invest* **84**: 451–457

Ovejera AA, Houchens DP and Barker AD (1978) Chemotherapy of human tumor xenografts in genetically athymic mice. *Ann Clin Lab Sci* **8**: 50–56

Park LS, Friend D, Gillis S and Urdal DL (1986) Characterization of the cell surface receptor for human granulocyte/macrophage colony-stimulating factor. *J Exp Med* **164**: 251–262

Rosen SW, Weintraub D and Aaronson SA (1980) Nonrandom ectopic protein production by malignant cells: direct evidence in vitro. *J Clin Endocrinol Metabol* **50**: 834–841

Rowe JM and Rapoport AP (1992) Hemopoietic growth factors: a review. *J Clin Pharmacol* **32**: 486–501

Russell PJ, Raghavan D, Philips J and Wills EJ (1988*a*) The biology of urothelial cancer. In *The Management of Bladder Cancer*, Raghavan D. (ed.), pp 1–41. Edward Arnold Publishers: Maryland, USA

Russell PJ, Wills EJ, Philips J, Wass J, Jelbart M, Gregory P and Raghavan D (1988*b*) Features of squamous and adenocarcinoma in the same cell in a xenografted human transitional cell carcinoma: evidence of a common histogenesis? *Urol Res* **16**: 79–84

Russell PJ, Jelbart M, Wills E, Singh S, Wass J, Wotherspoon J and Raghavan D (1988*c*) Establishment and characterization of a new human bladder cancer cell line showing features of squamous and glandular differentiation. *Int J Cancer* **41**: 74–82

Sato K, Terada K, Sugiyama T, Sugiyama T, Masuda H, Kakinuma H and Kato T (1994) Granulocyte colony-stimulating factor produced by bladder carcinoma of a patient with leukemoid reaction did not affect proliferation of the tumor cells. *J Urol* **151**: 1687–1690

Serve H, Steinhauser G, Oberberg D, Flegel WA, Northoff H and Berdel W (1991) Studies on the interaction between interleukin 6 and human malignant nonhematopoietic cell lines. *Cancer Res* **51**: 3862–3866

Shimamura K, Fujimoto J, Hata J-I, Akatsuka A, Ueyama Y, Watanabe T and Tamaoki N (1990) Establishment of specific monoclonal antibodies against recombinant human granulocyte colony-stimulating factor (hG-CSF) and their application for immunoperoxidase staining of paraffin-embedded sections. *J Histochem Cytochem* **38**: 283–286

Sires C, Neely S and Skinner D (1986) Leukemoid reaction in a patient with bladder and prostatic cancer. *J Urol* **135**: 366–367

Smith K, Fennelly JA, Neal DE, Hall RR and Harris AL (1989) Characterization and quantitation of the epidermal growth factor receptor in invasive and superficial bladder tumors. *Cancer Res* **49**: 5810–5815

Souza LM, Boone TC, Gabrilove J, Lai PH, Zsebo KM, Murdock DC, Chazin VR, Bruszewski J, Lu H, Chen KK, Barendt J, Platzer E, Moore MAS, Mertelsmann R and Welte K (1986) Recombinant human granulocyte colony-stimulating factor: effects on normal and leukemic myeloid cells. *Science* **232**: 61–65

Tachibana M (1982) Studies on cellular adhesiveness in five different culture cell lines derived from carcinoma of the bladder. *Keio J Med* **31**: 127–148

Tachibana M, Deguchi N, Baba S, Jitsukawa S, Hata M and Tazaki H (1992) Bromodeoxyuridine and deoxyribonucleic acid bivariate analysis in human renal cell carcinoma. Does flow cytometric determination predict malignant potential or prognosis of patients with renal cell carcinoma? *Am J Clin Pathol* **97**: s38–47

Tomonaga M, Golde DW and Gasson JC (1986) Biosynthetic (recombinant) human granulocyte–macrophage colony-stimulating factor: effects on normal bone marrow and leukemia cell lines. *Blood* **67**: 31–36

Vellenga E, Young DC, Wagner K, Wiper D, Ostapovicz D and Griffin JD (1987) The effects of GM-CSF and G-CSF in promoting growth of clonogenic cells in acute myeloblastic leukemia. *Blood* **69**: 1771–1776

Welte K, Platzar E, Lu L, Gabriloua JL, Levi E, Mertelsmann R and Moore MA (1985) Purification and biochemical characterization of human pluripotent haematopoietic colony-stimulating factor. *Proc Natl Acad Sci USA* **82**: 1526–1530

Wetzler M, Estrov Z, Talpaz M, Markowitz A, Gutterman JU and Kurzrock R (1993) Granulocyte–macrophage colony-stimulation factor as a cause of paraneoplastic leukemoid reaction in advanced transitional cell carcinoma. *J Intern Med* **234**: 413–420

Workman P, Balmain A, Hickman JA, McNally NJ, Mitchison NA, Pierrepoint CG, Raymond R, Rowlatt C, Stephens TC and Wallace J (1988) UKCCCR guidelines for the welfare of animals in experimental neoplasia. *Br J Cancer* **58**: 109–113

Young DC and Griffin JD (1986) Autocrine secretion of GM-CSF in acute myeloblastic leukemia. *Blood* **68**: 1178–1181

Zinzor SN, Svet-Moldavcky GJ, Fogh J, Mann PE, Arlin Z, Iliescu K and Holland JF (1985) Elaboration of granulocyte–macrophage colony-stimulating factor by human tumor cell lines and normal urothelium. *Exp Hematol* **13**: 574–580

*© Cancer Research Campaign 1997*